# Insulin Analogs and Cancer: A Note of Caution

**DOI:** 10.3389/fendo.2014.00079

**Published:** 2014-05-26

**Authors:** Joseph A. M. J. L. Janssen, Aimee J. Varewijck

**Affiliations:** ^1^Division of Endocrinology, Department of Internal Medicine, Erasmus MC, Rotterdam, Netherlands

**Keywords:** insulin, insulin analogs, IGF-I receptor, insulin receptor-A, insulin receptor-B, cancer, hyperglycemia

## Abstract

In view of the lifelong exposure and large patient populations involved, insulin analogs with an increased mitogenic effect in comparison to human insulin may potentially constitute a major health problem, since these analogs may possibly induce the growth of pre-existing neoplasms. At present, the available data suggest that insulin analogs are safe. In line with these findings, we observed that serum of diabetic patients treated with insulin analogs, compared to that of diabetic patients treated with human insulin, did not induce an increased phosphorylation of tyrosine residues of the insulin-like growth factor-I receptor (IGF-IR). However, the classical model of the IGF-IR signaling may be insufficient to explain (all) mitogenic effects of insulin analogs since also non-canonical signaling pathways of the IGF-IR may play a major role in this respect. Although phosphorylation of tyrosine residues of the IGF-IR is generally considered to be the initial activation step within the intracellular IGF-IR signaling pathway, it has been found that cells undergo a signaling switch under hyperglycemic conditions. After this switch, a completely different mechanism is utilized to activate the mitogenic (mitogen-activated protein kinase) pathways of the IGF-IR that is independent from tyrosine phosphorylation of the IGF-IR. At present it is unknown whether activation of this alternative intracellular pathway of the IGF-IR occurs during hyperglycemia *in vivo* and whether it is stronger in patients treated with (some) insulin analogs than in patients treated with human insulin. In addition, it is unknown whether the insulin receptors (IRs) also undergo a signaling switch during hyperglycemia. This should be investigated in future studies. Finally, relative overexpression of IR isoform A (IR-A) in (pre) cancer tissues may play a key role in the development and progression of human cancers during treatment with insulin (analogs). Further studies are required to unravel whether the IR-A is involved in the development of cancers and whether, in this respect (some) insulin analogs differ from human insulin.

## Introduction

Insulin analogs have been developed in an attempt to achieve a more physiological replacement therapy of insulin, thereby achieving a better glycemic control. However, structural modifications of the insulin molecule may also result in altered binding affinities and activities to the insulin-like growth factor-I receptor (IGF-IR). As a consequence, insulin analogs may (theoretically) have an increased mitogenic action compared to human insulin. In view of the lifelong exposure and large patient populations involved, insulin analogs with increased mitogenic effects in comparison to human insulin may constitute a major health problem, since these analogs could induce the growth of pre-existing neoplasms.

In 2009, several large observational studies suggested that use of insulin analogs and especially the use of insulin glargine was associated with an increased risk of cancer (1–4). The American Food and Drug Agency (FDA) concluded that the evidence presented was inconclusive due to the limitations in how these studies were designed, carried out, and in the data available for analysis [Early Communication about Safety of Lantus (insulin glargine); http://www.fda.gov/Drugs/DrugSafety/PostmarketDrugSafetyInformationforPatientsandProviders/DrugSafetyInformationforHeathcareProfessionals/ucm169722.htm did raise concerns as to whether or not insulin analogs promote cell proliferation and growth of (subclinical) neoplasms.

## Structural and Functional Overlap between Insulin and Insulin-Like Growth Factor-I

Very early in the evolution insulin and IGF-I emerged from a common molecule called proto-insulin (5). As a consequence of this common background there is a high homology in the molecular structure between insulin and IGF-I (5, 6). As insulin and IGF-I probably arose during evolution by gene duplication, there is the hypothesis that the insulin receptor (IR) and IGF-IR were also created by gene duplication of a common precursor receptor molecule (7). Due to the common evolutionary background, the molecular structure of the IR and the IGF-IR probably show high homology (7). Both consist out of two alpha subunits and two beta subunits, which are connected by disulfide bounds. Both receptors contain tyrosine kinase domains. Against their common background, it may not seem unexpected that both the IR and the IGF-IR share intracellular signaling pathways, which are involved in mitogenic and metabolic actions (8). Both insulin and IGF-I stimulate cell proliferation increasing DNA and RNA synthesis and by inhibiting apoptosis (9). In addition, both insulin and IGF-I effectuate metabolic effects, stimulate glucose uptake and protein synthesis, and inhibit breakdown of fat (9). Binding of human insulin to the IR primarily influences metabolic effects, although in some circumstances and in some cells it also induces growth promoting effects (10). In contrast, binding of IGF-I to the IGF-IR, primarily stimulates cell growth, but in some circumstances and in some cells, also stimulates metabolic effects (10).

*In vitro*, it has been found that insulin binds to the IGF-IR with low affinity and IGF-I binds to the IR with low affinity (10). However, the question remains whether this latter phenomenon also occurs *in vivo*.

**Figure 1:**
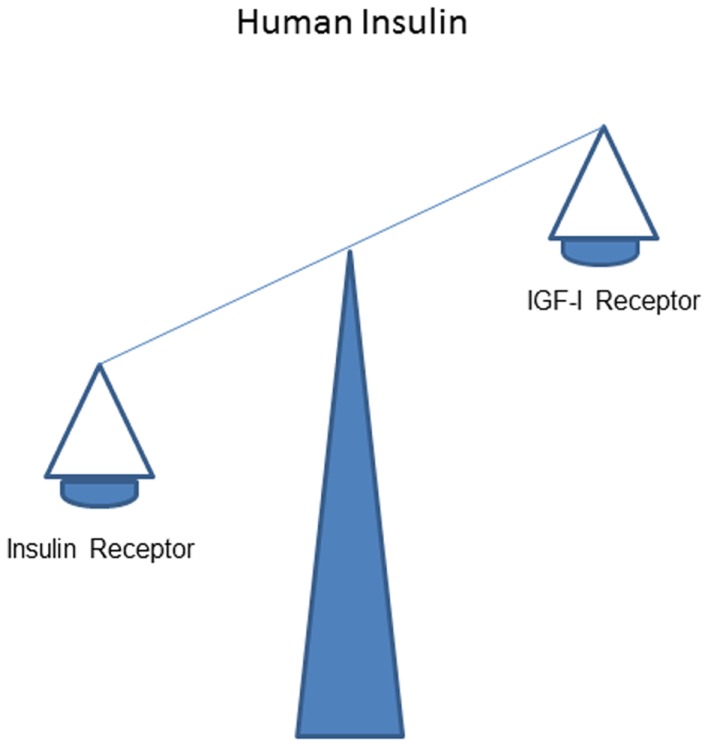
**Overall effects of human insulin on the insulin receptor dominate over that on the IGF-I receptor**.

It is thought that *in vivo* stimulating effects of human insulin on the IR normally dominate over that on the IGF-IR (Figure 1).

## Are Stimulating Effects of Insulin Analogs to the Insulin Receptor and the IGF-I Receptor Comparable to Human Insulin?

**Figure 2:**
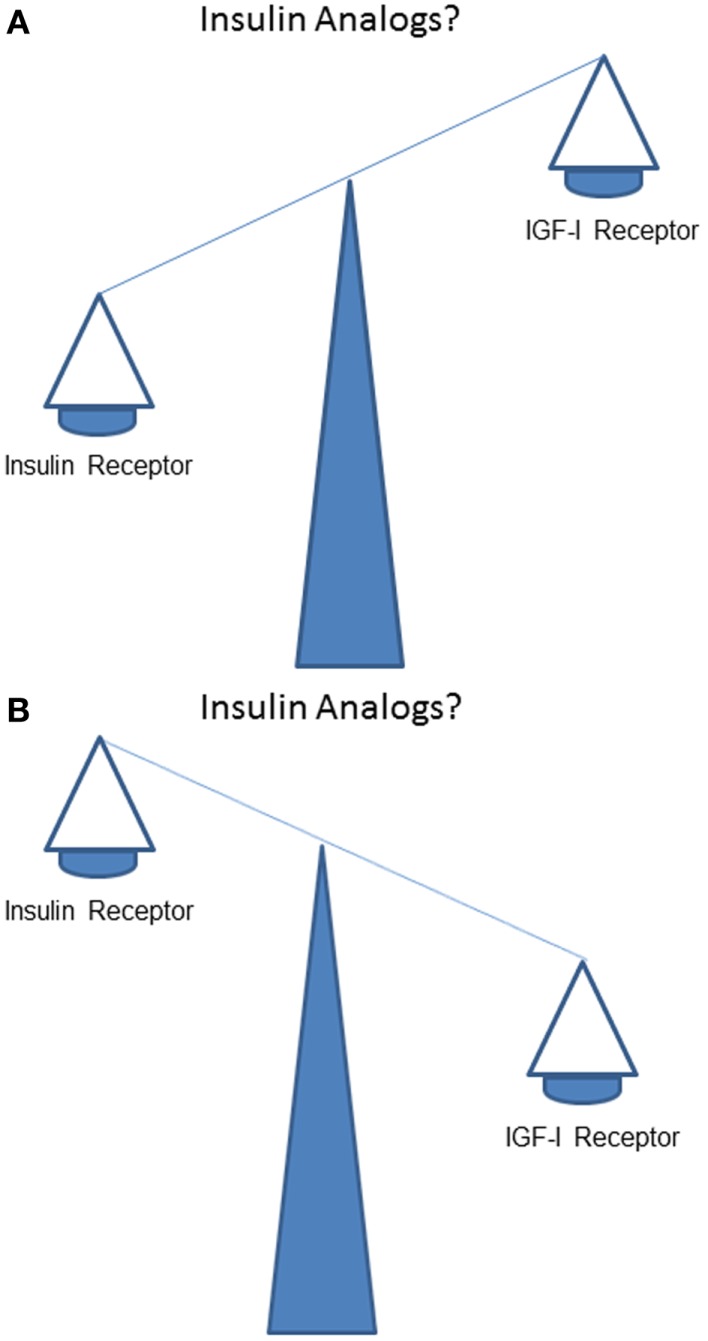
**What is the balance of effects of insulin analogs on the insulin receptor compared to the IGF-I receptor?** Do stimulating effects of insulin analogs on the insulin receptor dominate over the stimulating effects on the IGF-I receptor (like human insulin) **(A)**? Or, do stimulating effects of insulin analogs on the IGF-I receptor (in contrast to human insulin) dominate over stimulating effects of insulin analogs on the insulin receptor **(B)**?

How does stimulation of the IR by insulin analogs compare to stimulation of the IGF-IR *in vivo*? Does, like human insulin, IR stimulation dominate over IGF-IR stimulation (Figure 2A)? Or, alternatively and in contrast to human insulin, does IGF-IR stimulation dominate over IR stimulation (Figure 2B)?

Currently, there are three rapid-acting insulin analogs (insulin lispro, insulin aspart, and insulin glulisine) and three long-acting insulin analogs (insulin glargine, insulin detemir, and insulin degludec) commercially available. For insulin glargine (11), a slightly lower affinity for the IR but a significant higher affinity for the IGF-IR has been reported. For insulin aspart, the affinity for the IR and the IGF-IR have been reported to be similar to that of human insulin (12, 13). For insulin glulisine, compared to human insulin, a similar or even slightly less binding affinity for the IR has been found, while the IGF-IR binding affinity has been reported to be significantly lower than that of human insulin (14). *In vitro* studies show decreased IR binding affinity for insulin glargine compared to human insulin while the IGF-IR binding affinity *in vitro* has been reported to be stronger for insulin glargine than for human insulin (12, 15). For insulin detemir, compared to human insulin, the affinity for the IR *in vitro* has been found to be reduced, while the IGF-IR binding affinity has been reported to be significantly reduced as well as increased (11, 16). To date information on insulin degludec is limited. It has been reported that IR binding of insulin degludec is comparable to human insulin *in vitro*, while its affinity for the IGF-IR is low (17).

Thus, *in vitro*, all at present commercially available insulin analogs have equal or lower affinities for the IR than human insulin suggesting no important role of the IR in mitogenesis. At the same time, it has been suggested that especially insulin analogs with an increased affinity for the IGF-IR are more mitogenic than human insulin (13). In accordance with these data, cell models that permit comparisons of the activity of insulin to that of insulin analogs indicate that only minor differences exist between insulin and short-acting analogs (16). By contrast, some long-acting analogs activate the mitogenic signaling pathway more effectively than insulin and cause increased cell proliferation (16). However, the biological response of a target cell is not only determined by the affinity of the insulin analog–receptor interaction, but also by a number of other factors such as the concentration of receptors on the target cells and the concentration of an insulin analog. Interestingly, it has been reported that *in vivo* during treatment with insulin detemir and the recently introduced insulin degludec, relatively high circulating concentrations are achieved (18). Although a large fraction of these two insulins is bound to albumin, there is yet no clear information on the plasma concentrations of the free moiety for these two insulin preparations (18).

## Effects of Insulin Analogs Assessed by the IGF-I Kinase Receptor Activation Assay

In order to gain more insight into the possible growth promoting effects of insulin analogs, we have used the so-called IGF-I kinase receptor activation (KIRA) assay. The IGF-I-KIRA assay uses a human embryonic kidney cell line (293 EBNA) highly transfected with cDNA encoding the full-length of the IGF-IR gene (19). The IGF-I KIRA assay is capable of quantifying IGF-I bioactivity by measuring IGF-I-induced receptor-phosphorylation after binding of IGF-I or other ligands to the IGF-IR (19). As above discussed the IGF-IR contains two alpha subunits, two beta subunits, which contain both a so called tyrosine kinase domain. Binding of IGF-I or another ligand to the IGF-IR causes phosphorylation of tyrosine residues in the beta units, which is generally considered to be the initial step in activation of the intracellular IGF-IR signaling pathway. This first step of intracellular signaling can be quantified in a time resolved fluorometer [for more details of this method see Ref. (20)].

By using the IGF-I KIRA assay, we compared IGF-IR activation *in vitro* induced by human insulin, two short-acting insulin analogs (insulin aspart and insulin lispro) and two long-acting insulin analogs (insulin glargine and insulin detemir). Overall, short-acting insulin analogs did not differ substantially in activating the IGF-IR from human insulin. Insulin lispro was slightly more potent in activating the IGF-IR than human insulin and insulin aspart, only reaching statistical significance at 100 nM (21). The two long-acting insulin analogs differed substantially from each other in activating the IGF-IR. At concentrations above 1 nmol/L, insulin glargine was more potent in IGF-IR activation than human insulin and insulin detemir (21). However, at more physiological concentrations (i.e., below concentrations of 1 nmol/L), no differences in IGF-IR activation were observed between insulin glargine, human insulin, and insulin detemir (21). The IGF-IR activation induced by insulin glargine was significantly lower compared to pure IGF-I over the whole range of concentrations tested. Thus our *in vitro* experiments showed that insulin glargine was able to activate the IGF-IR more strongly than human insulin and insulin detemir, yet still significantly less strong than IGF-I.

By using the same IGF-I KIRA assay in another *in vitro* experiment, we compared IGF-IR activation induced by insulin glargine and insulin NPH. Insulin glargine was more potent in IGF-IR activation than NPH insulin at 10–100 nmol/L (22). Again we found no differences in IGF-IR activation between insulin glargine and NPH insulin at more physiological concentrations (i.e., below concentrations of 1 nmol/L) (22).

Next we studied serum samples of 104 patients with type 2 diabetes from the LANMET study (23). In the LANMET study, diabetic subjects, who were poorly controlled under metformin monotherapy, were randomized to receive either metformin with insulin glargine or metformin with NPH insulin (23). We measured IGF-I bioactivity with the IGF-IR KIRA assay in fasting samples at two time points: at baseline and after 36 weeks of combined metformin and insulin therapy. Importantly, in the 36 weeks of follow-up metabolic control improved and overall HbA1c decreased from 75 to 53 mmol/mol (9–7%). IGF-I bioactivity significantly decreased after 36 weeks of treatment and was slightly lower than a non-diabetic control group (22). In this respect, there were no differences in circulating IGF-I bioactivity and metabolic control between subjects treated with insulin glargine and subjects treated with NPH (22).

## How to Explain the Discrepancy between the *In vitro* and *Vivo* Results with the IGF-I KIRA Assay?

As discussed above, the *in vitro* findings leading to the hypothesis of an increased growth promoting activity on the IGF-IR by insulin glargine could not be supported by our *in vivo* data. What could explain the observed differences in IGF-I bioactivity after administration of insulin glargine during *in vitro* and *in vivo* conditions?

There are at least *three not mutually exclusive explanations* for our findings:

(1) *In vivo* insulin glargine did not reach concentrations at which we and others have observed differences in IGF-IR activation *in vitro* compared to human insulin.
(2) It should be stressed that in the LANMET study all subjects were treated with metformin. Metformin appears to have pleiotropic mechanisms of action, including increasing peripheral insulin sensitivity (24). Since an inverse relationship between insulin sensitivity and circulating IGF-I bioactivity has been found (25–27), the metformin-induced increase in peripheral insulin sensitivity may have contributed to the observed lower circulating IGF-I bioactivity during insulin glargine treatment.
(3) Furthermore, after subcutaneous injection, insulin glargine is partially degraded into two bioactive products (M1 and M2) (28). After 36 weeks of insulin glargine treatment, we found that plasma M1 concentrations had increased from undetectable to 1.5 ng/mL, while insulin glargine itself and M2, the other metabolite of insulin glargine, remained undetectable (29). The M1 degradation product has previously been shown to have considerable less mitogenic potency than insulin glargine itself (28). In line with this finding, we found that, *in vitro*, compared to NPH insulin, M1 activated the IGF-IR similarly (29).

Recently, the prospective ORIGIN trial provided evidence that low dose insulin glarigine (median 0.40 units/kg), when used to target normal fasting plasma glucose levels for more than 6 years, had a neutral effect on the development of cancers (30). Thus all these data are reassuring and suggest that insulin glargine compared to human insulin (at least in low doses) does not increase IGF-IR signaling *in vivo*.

Nevertheless, in our opinion the debate about long-term safety of insulin analogs is still not closed. Like all *in vitro* systems, the IGF-I KIRA assay does not mimic the exact *in vivo* conditions. Vigneri et al. have suggested that long-acting insulin analogs have a prevalent activation of the extracellular regulated kinase (ERK) pathway (the mitogenic pathway) rather than the AKT pathway (which is considered the metabolic pathway) (13). Although with the IGF-I KIRA assay, phosphorylation of the tyrosine residues within the ß-subunits of the IGF-IR is quantified, it cannot be assessed whether or not activation *in vivo* of the IGF-IR by an insulin analog results (like human insulin) in a balanced metabolic and mitogenic activity at cellular level.

**Figure 3:**
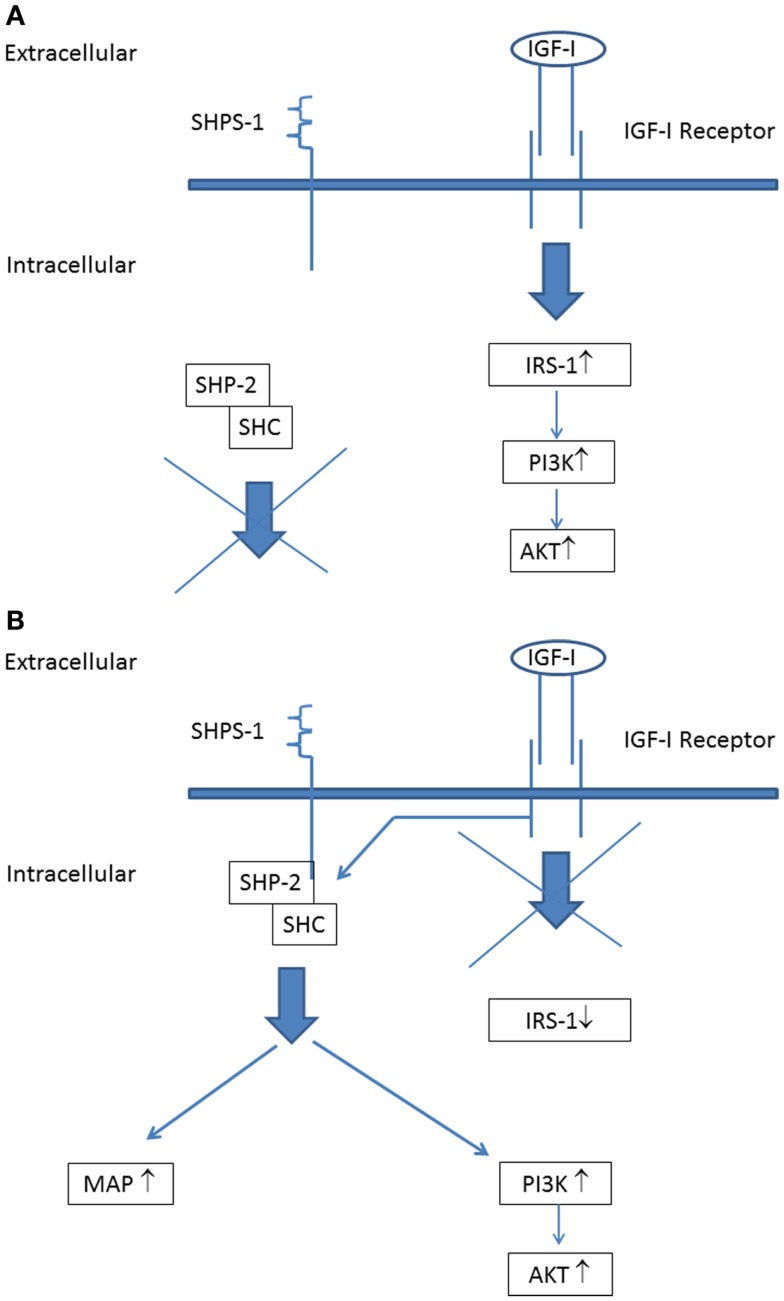
**The IGF-I receptor signaling pathways during normoglycemia:** (A) stimulation of the IGF-I receptor leads to phosphorylation of insulin receptor substrate-1 (IRS-1) that couples IGF-I receptor stimulation to downstream activation of the PI-3-kinase (“metabolic”) signaling pathway. **(B)** IGF-I receptor signaling pathways during hyperglycemia: stimulation of the IGF-I receptor leads not phosphorylation of IRS-1. Instead of this, IGF-IR linked signaling occurs via its ability to phosphorylate SHPS-1, which results in assembly of a SHPS-1 signaling complex which leads to stimulation of both the PI-3 kinase (“metabolic”) signaling pathway and MAP kinase (“mitogenic”) signaling pathway [Figure modified from Clemmons et al. (32); see also text].

A further important point to address is that all serum samples of the diabetic subjects from the LANMET study were studied with the IGF-I KIRA assay *in vitro* under normoglycemic conditions (5.5 mmol/L). Hyperglycemia ensures a high glucose supply for cells favoring anabolic metabolism to fuel tumor growth and this has been suggested (at least partly) to explain the increased cancer risk associated with diabetes (31). However, hyperglycemia may also change IGF-IR signaling. Clemmons et al. have shown that, under normoglycemic conditions, stimulation of the IGF-IR expressed on vascular smooth muscle cells and vascular endothelial cells only activates IRS-1 leading to stimulation of the “metabolic” (phosphoinositide 3) PI-3 kinase pathway, but not to stimulation of the “mitogenic” (mitogen-activated protein) MAP kinase pathway (32) (Figure 3A). However, they also found that, following exposure to hyperglycemia, cells undergo a signaling switch *in vitro* leading to an entirely different mechanism to activate both the “metabolic” (PI-3 kinase) and “mitogenic” (MAP) pathways of the IGF-IR (32). This signaling switch leads to increased proliferation and migration.

Activation of this alternative signaling mechanism occurs in hyperglycemic conditions independent from tyrosine phosphorylation of the IGF-IR: IGF-IR linked signaling occurs via its ability to phosphorylate SH2 domain-containing protein tyrosine phosphatase substrate-1 (SHPS-1), which results in assembly of a SHPS-1 signaling complex which leads to both the PI-3 kinase and MAP kinase activation (32) (Figure 3B). Thus these findings show that under hyperglycemic conditions stimulation of cell growth may occur *independently from tyrosine phosphorylation of the IGF-IR*.

Clemmons et al. postulated that activation of this alternative signaling pathway is directly linked to the pathophysiologic processes that are involved in the pathogenesis of diabetic complications such as diabetic retinopathy and atherosclerosis (32). Since cancer can be considered as a “new complication” of diabetes (33) while there is also a clear relationship between hyperglycemia and incidence of cancer in type 2 diabetes and the metabolic syndrome (34–38), we think that this hypothesis may be extended and that it is worthwhile to investigate whether hyperglycemia-induced activation of this alternative intracellular pathway of the IGF-IR is also involved in the development of cancer in diabetes. At present it is unclear whether, during hyperglycemic conditions, activation of this alternative intracellular pathway of the IGF-IR is different for (some) insulin analogs compared to human insulin. This should be investigated in future studies.

Insulin-like growth factor-I receptor is traditionally described as an ON/OFF system, with ligand stabilizing the ON state by exclusively kinase-dependent signaling activation (39). In fact, the IGF-I KIRA assay is based on the idea that autophosphorylation of tyrosine residues within the kinase domain of the IGF-IR is the first step and essential for subsequent activation of the intracellular signaling pathways. However, more recently it was suggested that the IGF-IR also behaves like a functional receptor tyrosine kinase/G-protein related coupled receptor hybrid “borrowing” components of G-protein coupled receptor signaling (39). In addition to the above described classical kinase pathway, IGF-IR activity and its biological effects are further controlled by a variety of adaptor proteins/signaling proteins through IGF-IR posttranslational modifications including tyrosine and serine phosphorylation, dephosphorylation, ubiquitination, and sumoylation (39). In the light of the complexity of the downstream pathways activated by the IGF-IR, it is conceivable that (some) insulin analogs may use different post-receptor signaling pathways than human insulin.

## Potential Role of IR-A in the Development of Cancer

Mitogenic effects of insulin may also occur via increased stimulation of the IR (40). In addition, Hansen et al. have shown that increased mitogenic potency of insulin analogs may also result from slow ligand dissociation from the IRs (40). The IR may be produced as A or B isoforms (IR-A and IR-B, respectively) and both forms show different biological characteristics (41, 42). The IR-B is considered the typical insulin target tissue receptor in the muscles, liver and fat cell, and mainly involved in the insulin-mediated metabolic effects (43). The IR-A is expressed ubiquitously, but is predominantly expressed in the central nervous system and in hematopoietic cells (44). Interestingly, especially the IR-A is also overexpressed in many human cancers and IR-A has stronger mitogenic activity than the IR-B.

The IR-A has the peculiar characteristic not only to be activated by insulin but also by IGF-II, and although to a lesser extent to IGF-I (42, 45). Very recently, an important role for the IR-A in the development of cancer has been suggested (16). This opens the possibility that the IR-A holds an important position in the stimulation of cancer cell proliferation in response to insulin and insulin analogs (42, 43). Relative IR-A and IR-B expression may vary in a tissue-specific manner and inter-individual differences in the levels of proteins of the IR-A and IR-B may function as a critical determinant of the mitogenic potency of insulin and insulin analogs. In most cancer types, the IR-A/IR-B ratio is changed; upregulation of IR-A has been reported in breast, ovarian, colon and thyroid cancer cell lines, and human tumors (42, 43). Relative overexpression of IR-A may play a key role in the development and progression of human cancers after starting treatment with insulin (analogs).

## Signaling Pathways of the Insulin Receptors

Stimulation of the IRs activates at least two important different signaling pathways (one involving MAP kinases, one involving PI-3 kinase) (42). A third pathway may be translocation of complexes of insulin or insulin analogs bound the IRs to the nucleus of the cell (45, 46). Although the role of insulin internalization and translocation to the nucleus is still controversial, there is substantial evidence to support a role in cellular mediated responses induced by insulin. In favor of this latter possibility many studies indicate that nuclear translocation of various growth factors and hormones plays an important role in cell proliferation or DNA synthesis (46). Further studies are required to reveal which signaling pathways are actually involved in the different effects and whether in this respect (some) insulin analogs differ from human insulin.

There is probably a considerable crosstalk between the IR-A, IR-B, and IGF-IR mediated functions at the receptor and post-receptor level and the final effects are due to a combination of IGF-IR and IRs-mediated processes. It has been suggested that the IR and IGF-IR act at identical portals to the regulation of gene expression, with differences between insulin and IGF-I effects due to a modulation of the signal created by the specific ligand–receptor interactions (47). In addition, it has been also suggested that various ligands acting through the same receptor may activate different patterns of end-point cellular effects (“differential signaling”) (48). As a consequence, it is almost impossible in most *in vitro* cell lines to study and disentangle the individual effects of insulin and insulin analogs on the IR-isoforms.

## Are the Insulin Analog-Mediated Effects on the IR-A Different from those of Human Insulin?

Due to the above discussed considerable crosstalk between the IR-A, IR-B, and IGF-IR, there is at present only limited information on the interaction between the insulin analogs on the two IR-isoforms. Sciacca et al. compared several insulin analogs for mitogenic effects in engineered cells expressing only the IR-A or IR-B, and found that relative to human insulin, long-acting insulin analogs like insulin glargine and insulin detemir strongly activated the ERK pathway and cell proliferation via the IR-A (16).

In our laboratory, we have developed KIRA assays specific for both the IR-A and the IR-B (46). Both assays are capable of quantifying IR stimulating activity by measuring induced receptor tyrosine kinase activation in individual serum samples. By using KIRA assays specific for the IR-A and the IR-B, we tested (*in vitro*) whether short-and long-acting insulin analogs differed from human insulin in their potency to activate the IR-A and IR-B. In line with a previous study by Kurtzhals et al., we found that short-acting insulin analogs (insulin lispro and insulin aspart) did not differ substantially from human insulin, nor from each other in activating either receptors (15, 21). When comparing long-acting insulin analogs with NPH insulin, we observed that in a concentration range of 10–100 nmol/L insulin glargine and M2 were more potent than NPH insulin in activating IR-A and IR-B while M1 activated IR-A and IR-B similarly compared to NPH insulin (29). Thus *in vitro* at supraphysiological concentrations, insulin glargine, and its metabolite M2 were more potent in activating both IR-isoforms compared to human insulin.

To investigate whether our *in vitro* results could be extrapolated to the *in vivo* situation, we compared serum induced IR-A and IR-B activation (by using an IR-A and IR-B specific KIRA assay, respectively) of type 2 diabetes patients treated with relatively high doses of insulin glargine or NPH insulin (29). Serum IR-A and IR-B bioactivity did not differ between patients treated with insulin glargine or NPH insulin (29). Our results did not support the idea that treatment with insulin glargine in type 2 diabetes leads to a stronger stimulation of the IRs than NPH insulin.

In subjects treated with insulin glargine therapy, we also measured insulin glargine, M1, and M2 concentrations in plasma (29). Only M1, but not insulin glargine nor M2 could be detected in the plasma of these subjects (29). These latter findings may explain why we could not find differences in potency of serum to activate the IR-A and IR-B between subjects treated with insulin glargine or NPH insulin.

Nevertheless, we observed a positive relationship between insulin dose and serum induced IR-A activation in both the insulin glargine and NPH insulin treatment groups (29). This suggests that subjects treated with relatively high doses of insulin (analog) did have the strongest activation of the IR-A. At present it is unclear whether this has any consequences in daily clinical practice and whether subjects using relatively high daily doses of insulin (analogs) have an increased risk to develop cancer (see below). Appropriately designed prospective observational studies comparing diabetic patients treated with “low” and “high” doses of an insulin analog may add further useful information and shed more light onto this important issue.

## Is the Risk of Cancer Directly Related to the Dose of Insulin (Analogs) and/or Metabolic Control?

The debate continues to the role of insulin (analogs) when high (supraphysiological) doses of insulin (analogs) are used in the treatment of type 2 diabetes in an attempt to achieve normoglycemia.

It is important to realize that in routine daily clinical practice there is a considerable delay in initiation of insulin therapy after failure of oral glucose-lowering agents in patients with type 2 diabetes (49). But, even after starting insulin (analog) treatment, a considerable number of subjects will still not achieve target HbA1c levels ≤7% (50). In addition, type 2 diabetes is a progressive disease characterized by worsening of glycemia even after starting insulin (analog) treatment. As a consequence, insulin (analog) doses usually increase over time in order to achieve good metabolic control (51).

**Figure 4:**
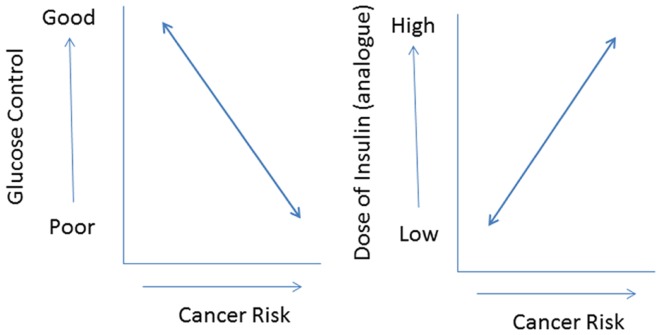
**The use of insulin (analogs) may have dual effects in type 2 diabetes: decreasing cancer risk by improving metabolic control (left) but simultaneously increasing cancer risk because of its (dose-dependent) effects on cell growth and proliferation (right)**. In this scenario especially subjects with poor metabolic control treated with relatively high pharmacological doses of insulin analogs will be at an increased risk for cancer.

In addition, most commercially available insulin analogs often show less metabolic activity than human insulin *in vitro* (11). If this is translated into an *in vivo* situation, it is very plausible that compared to human insulin higher doses of an insulin analog are required to attain a comparable metabolic control. As a consequence, *in vivo* relatively high(er) concentrations of insulin analogs will be present in the circulation during treatment. However, relatively high(er) concentrations of an insulin analog may not only improve metabolic control but also increase cancer risk by dose-dependent effects on cellular differentiation, growth, and proliferation. Thus use of insulin analogs may have *dual effects* in type 2 diabetes: decreasing cancer risk by improving metabolic control but simultaneously increasing cancer risk, because of its dose-dependent effects on cell growth and proliferation (37) (Figure 4). In this scenario, the effects of insulin analogs on cancer risk are directly related to glucose control: subjects with poor metabolic control treated with relatively high (pharmacological) doses of an insulin analog especially run an increased risk for cancer.

As discussed above, upon exposure to hyperglycemia, cells may also undergo a signaling switch of IGF-IR and use alternative post-receptor signaling mechanisms (32). Whether this latter phenomenon also occurs for the IRs following exposure to hyperglycemia is at present unknown and warrants further investigation.

In conclusion, there is a complex relationship between the use of insulin analogs, hyperglycemia, and cancer risk. In view of the lifelong exposure and large patient populations involved, insulin analogs with an increased mitogenic effect may potentially constitute a major health problem when inducing the growth of pre-existing neoplasms. The available data so far suggest at present that insulin analogs are safe.

Risk of cancer may be directly related to the dose of insulin (analogs) and/or effects on metabolic control. Upon exposure to hyperglycemia, cells may undergo a signaling switch. After this switch, independent from tyrosine phosphorylation of the IGF-IR, an entirely different mechanism is utilized to activate the mitogenic (MAP) pathways of the IGF-IR. At present it is unclear whether activation of this alternative intracellular pathway of the IGF-IR under hyperglycemic conditions is stronger during treatment with (some) insulin analogs compared to human insulin. In addition, whether a similar signaling switch also occurs for the IRs under hyperglycemic conditions is at present unclear. This should be investigated in future studies.

Finally, a relative overexpression of IR-A in (pre) cancer tissues may play a key role in the development and progression of cancers. *In vitro*, we observed that high doses of insulin analogs/insulin did have the strongest activation of the IR-A. However, at present it is unclear whether this has any consequences in clinical practice or whether insulin analogs differ from human insulin in this respect. Well-conducted and appropriately designed prospective observational studies comparing diabetic patients treated with “low” and “high” doses of an insulin analog should be started to shed more light onto this important issue.

## Author Contributions

Both Joseph A. M. J. L. Janssen and Aimee J. Varewijck researched data, wrote manuscript, and reviewed/edited the manuscript.

## Conflict of Interest Statement

The authors declare that the research was conducted in the absence of any commercial or financial relationships that could be construed as a potential conflict of interest.
